# Two New SGI1-LK Variants Found in Proteus mirabilis and Evolution of the SGI1-HKL Group of *Salmonella* Genomic Islands

**DOI:** 10.1128/mSphere.00875-19

**Published:** 2020-03-04

**Authors:** Claire de Curraize, Eliane Siebor, Véronique Varin, Catherine Neuwirth, Ruth M. Hall

**Affiliations:** a Bacteriology Department, University Hospital Dijon, Dijon, France; b UMR 6249, Chrono-Environnement, Dijon, France; c School of Life and Environmental Sciences, The University of Sydney, Sydney, New South Wales, Australia; Escola Paulista de Medicina/Universidade Federal de São Paulo

**Keywords:** *Salmonella* genomic island 1, *Proteus mirabilis*, IS*26*, evolution, SGI1

## Abstract

Integrative mobilizable elements belonging to the SGI1-H, -K, and -L *Salmonella* genomic island 1 (SGI1) variant groups are distinguished by the presence of an alteration in the backbone (IS*1359* replaces 2.8 kb of the backbone extending from within *traN* [S005] to within S009). Members of this SGI1-HKL group have been found in Salmonella enterica serovars and in Proteus mirabilis. Two novel variants from this group, designated SGI1-LK1 and SGI1-LK2, were found in the draft genomes of antibiotic-resistant P. mirabilis isolates from two French hospitals. Both variants can be derived from SGI1-*Pm*GUE, a configuration found previously in another P. mirabilis isolate from France. SGI1-LK1 could arise via an IS*26*-mediated inversion in the complex class 1 integron that duplicated the IS*26* element and the target site in IS*6100*. SGI1-LK1 also has a larger 8.59-kb backbone deletion extending from *traN* to within S013 and removing *traG* and *traH.* However, SGI1-LK1 was mobilized by an IncC plasmid. SGI1-LK2 can be derived from a hypothetical progenitor, SGI1-LK0, that is related to SGI1-*Pm*GUE but lacks the *aphA1* gene and one copy of IS*26.* The integron of SGI1-LK2 could arise via deletion of DNA adjacent to an IS*26* and a deletion occurring via homologous recombination between duplicated copies of part of the integron 3′-conserved segment. SGI1-K can also be derived from SGI1-LK0. This would involve an IS*26*-mediated deletion and an inversion via homologous recombination of a segment between inversely oriented IS*26*s. Similar events can explain the configuration of the integrons in other SGI1-LK variants.

**IMPORTANCE** Members of the SGI1-HKL subgroup of SGI1-type integrative mobilizable elements have a characteristic alteration in their backbone. They are widely distributed among multiply antibiotic-resistant Salmonella enterica serovars and Proteus mirabilis isolates. The SGI1-K type, found in the globally disseminated multiply antibiotic-resistant Salmonella enterica serovar Kentucky clone ST198 (sequence type 198), and various configurations in the original SGI1-LK group, found in other multiresistant S. enterica serovars and Proteus mirabilis isolates, have complex and highly plastic resistance regions due to the presence of IS*26*. However, how these complex forms arose and the relationships between them had not been analyzed. Here, a hypothetical progenitor, SGI1-LK0, that can be formed from the simpler SGI1-H is proposed, and the pathways to the formation of new variants, SGI1-LK1 and SGI1-LK2, found in P. mirabilis and other reported configurations via homologous recombination and IS*26*-mediated events are proposed. This led to a better understanding of the evolution of the SGI1-HKL group.

## Abstract (precis)

Members of the SGI1-HKL subgroup of SGI1-type integrative mobilizable elements have a characteristic alteration in their backbone. They are widely distributed among multiply antibiotic-resistant Salmonella enterica serovars and Proteus mirabilis isolates. The SGI1-K type, found in the globally disseminated multiply antibiotic-resistant Salmonella enterica serovar Kentucky clone ST198 (sequence type 198), and various configurations in the original SGI1-LK group, found in other multiresistant S. enterica serovars and Proteus mirabilis isolates, have complex and highly plastic resistance regions due to the presence of IS*26*. However, how these complex forms arose and the relationships between them had not been analyzed. Here, a hypothetical progenitor, SGI1-LK0, that can be formed from the simpler SGI1-H is proposed, and the pathways to the formation of new variants, SGI1-LK1 and SGI1-LK2, found in P. mirabilis and other reported configurations via homologous recombination and IS*26*-mediated events are proposed. This led to a better understanding of the evolution of the SGI1-HKL group.

## INTRODUCTION

Members of the *Salmonella* genomic island 1 (SGI1) family are integrative mobilizable elements (IMEs) that contribute to the problem of multiple-antibiotic resistance (MAR) in Gram-negative bacteria, as they carry various sets of antibiotic resistance genes in a class 1 integron (1). They have been found so far in several Salmonella enterica serovars, Proteus mirabilis (1), and, more recently, Morganella morganii, Providencia stuartii, and Escherichia coli (2). SGI1 and its variants are inserted at the 3′-end of the chromosomal *trmE* (formerly *thdF*) gene and are mobilized specifically by IncA and IncC plasmids (1, 3–7).

SGI1, the first variant identified, was found in Salmonella enterica serovar Typhimurium and is made up of a 27.4-kb backbone containing 28 open reading frames (ORFs) from *int*_SGI1_ (S001) to *resG* (S027) and S044 and a 15-kb complex class 1 integron inserted upstream of the *resG* gene and flanked by a 5-bp duplication (ACTTG) (8). The complex class 1 integron harbors an *aadA2* cassette encoding streptomycin and spectinomycin resistance at the first *attI* site and a *blaP* (also known as *bla*_PSE-1_) cassette encoding resistance to penicillins at the second *attI* site (8, 9). Most variants (SGI1-A to SGI1-Z and others with various names) have differences in the class 1 integron, with cassette array exchanges or reduction to a simple integron via homologous recombination being the most common (1, 9–12).

**FIG 1:**
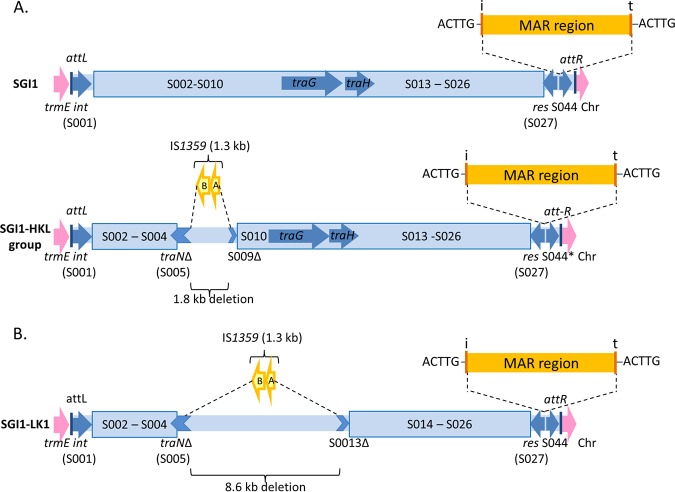
Schema of SGI1 variants. The chromosomal genes are in pink, the backbones of SGI1 variants are in blue, and the multiple-antibiotic-resistance (MAR) region and IS*1359* are represented in yellow. *attL* and *attR* are the left and right attachment sites, respectively. The position of the class 1 integron is indicated by the 5-bp duplication (ACTTG). (A) SGI1 and SGI1-HKL variant group. SGI1 has already been reported to be inserted at the 3′-end of the *trmE* gene upstream of the following chromosomal (Chr) genes: the *int2* gene (retron phage gene) in *S.* Typhimurium DT104, the *yidY* gene of S. enterica, and the *hipB* gene of Proteus mirabilis. The SGI1-H, -K, and -L groups were previously reported to be inserted at the 3′-end of the *trmE* gene upstream of the following chromosomal genes: the *yidY* gene in S. enterica, the *hipB* gene in P. mirabilis, and the permease gene in Morganella morganii. *, the 5′-end of S044 is deleted in SGI1-K variants. (B) SGI1-LK1 variant. The SGI1-LK1 variant is inserted at the 3′-end of the *trmE* gene upstream of the *hipB* gene of P. mirabilis.

Among these variants, there is a group of variants that have a characteristic alteration in the backbone (1, 13). This group includes SGI1-H, SGI1-K, SGI1-L (14–17), and variants derived from them (13, 18, 19) as well as SGI1-P and SGI1-Q, which appear to have arisen from SGI1-K (18, 20). In the backbone of these variants, the insertion sequence IS*1359* (or IS*Vch4*) has replaced 2.8 kb of the backbone extending from within *traN* (S005) to within S009 (Fig. 1A). This deletion does not abolish transfer (4, 6). However, the class 1 integrons of these variants are at the same position as in SGI1, suggesting that this alteration occurred after the acquisition of the class 1 integron. Here, this group is called the SGI1-HKL group.

SGI1-H, first reported in S. enterica serovar Newport, harbors a complex class 1 integron which differs from In104 in SGI1 only by the presence of the *aacCA5*-*aadA7* cassette array encoding aminoglycoside resistance at the first *attI* site instead of *aadA2* (14). This replacement probably resulted from a cassette array exchange via homologous recombination. As originally described, SGI1-L in *S.* Newport strain 00-4093 also harbors a complex class 1 integron, which includes the *dfrA15* gene encoding trimethoprim resistance at the first *attI* site (16). However, later, it was observed that the *aacCA5-aadA7* cassette array had not been lost and that an IS*26* composite transposon containing a major part of the SGI1-K integron (15) was also present, and SGI1-L was renamed SGI1-L1 (18). Since then, SGI1-L, as originally described, has been reported in Proteus mirabilis (21), and recently, the complete sequence of SGI1-L in Morganella morganii was released (22).

SGI1-K was first reported in S. enterica serovar Kentucky (15, 17). SGI1-K harbors a more complex resistance region. The integron contains the first cassette array of SGI1-H (*aacCA5*-*aadA7*) but is followed by a hybrid (Tn*501*/Tn*21*) *mer* module and fragments of different various transposons, Tn*1721* with *tetA*(A) encoding resistance to tetracyclines, Tn*5393* with the *strAB* streptomycin resistance genes, and Tn*2* containing *bla*_TEM-1b_ encoding a penicillinase (15, 17). This region also contains two copies of IS*26*, and one has promoted a deletion that removed a part of the integron and 292 bp from the 5′ end of S044. The presence of these two IS*26*s promotes further evolution of the resistance region by deletion and inversion of adjacent segments (23), and IS*26* can also promote the insertion of further resistance genes (24, 25). IS*26* action has generated many derivatives of SGI1-K, some of which were numbered, like SGI1-K2 to SGI1-K7 (13, 18, 19), and some of which were not (20). It can also remove a major part of the integron (SGI1-P1, -P2, -Q1, and -Q2) or completely remove it (SGI1-Q3), or it can delete a part of the adjacent backbone (13, 20). SGI1-K and deletion derivatives are found in the worldwide *Salmonella* Kentucky clone ST198 (sequence type 198) (15, 20, 26), as are the SGI1-P and SGI1-Q types (13). Another SGI1-K variant has been found in *S.* Newport (SGI1-K6) (18).

Although initially found in S. enterica serovars, SGI1 variants are also found in other species, indicating that interspecies transfer has occurred. Among other species, P. mirabilis has proven to be a rich source of novel variant forms (11, 19, 27). These include members of the SGI1-HKL group with a variety of names, SGI1-K7 (19), SGI1-*Pm*ABB, SGI1-*Pm*MAT, SGI1-*Pm*SCO (SGI1-H), SGI1-*Pm*VER, and SGI1-*Pm*GUE (27). SGI1-*Pm*GUE harbors all of the components found in SGI1-K and SGI1-L but has an additional IS*26* and *aphA1* gene (encoding kanamycin and neomycin resistance) that have combined with a preexisting IS*26* to generate Tn*4352*. Part of *tnpM* of Tn*21* is also present. This structure has the potential to explain the relationship between SGI1-H and -L as well as the derivation of SGI1-K.

Here, we report the complete sequences of two new variants of the SGI1-HKL group recovered from two clinical P. mirabilis isolates, *Pm*294MATLI and *Pm*144BOUSA, in France. SGI1-*Pm*GUE and a possible progenitor of SGI1-*Pm*GUE that lacks the *aphA1* region are examined as potential progenitors of these variants and of SGI1-K.

## RESULTS

### Characterization of the SGI1 variant in *Pm*294MATLI.

The P. mirabilis strain *Pm*294MATLI was isolated from pus of a breast wound of a patient hospitalized in Assistance Publique des Hôpitaux de Paris (APHP), Lariboisière Hospital, in Paris, France, in 2015. In addition to intrinsic resistance to doxycycline and colimycin, this strain was resistant to penicillins, kanamycin, streptomycin, spectinomycin, gentamicin, chloramphenicol, sulfonamides, and trimethoprim. The draft genome was determined and found to harbor the same resistance genes as SGI1-*Pm*GUE, namely, *aacCA5*, *aadA7*, *sul1*, *tetA*(A), *strAB*, *bla*_TEM-1b_, *aphA1*, *dfrA15*, *floR*, and *blaP*.

**FIG 2:**
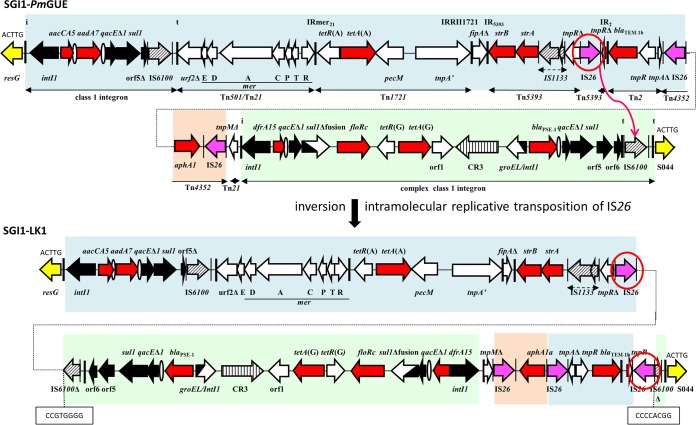
The MAR region of SGI1-*Pm*GUE and generation of the MAR region of SGI1-LK1. Horizontal arrows indicate genes and open reading frames (ORFs) with their transcriptional orientation. The segments with genes and ORFs present in SGI1-K are in blue, those present in SGI1-L are in green, and those corresponding to IS*26-aphA1* are in orange. Backbone genes are represented in yellow. Black arrows represent 5′ conserved segments and 3′ conserved segments of integrons. The thick vertical bars indicate the inverted repeats (IRs) of integrons (IRi) and transposons (IRt). Resistance genes are shown with red arrows, and ovals represent *attC* sites. The insertion sequences (ISs) are represented by hatched arrows and by purple arrows for IS*26*. The IS*26*s predicted to cause rearrangements are circled. Their IRs are represented by thin vertical bars.

An SGI1 variant was found at the 3′-end of the chromosomal *trmE* gene of *Pm*294MATLI and was assembled by PCR from 14 contigs (see Materials and Methods). It was 61.07 kb long (GenBank accession number MH734354), and like the SGI1-HKL variants, the backbone includes an IS*1359* element, with one end at the same position in *traN* as in the SGI1-HKL group. However, the deletion was larger, 8.57 kb in total, and extended from within *traN* to within S013, removing the *traG* and *traH* genes (Fig. 1B). The 41.02-kb integron found upstream of *resG* was flanked by the same 5-bp duplication (ACTTG) as in other SGI1 variants. Like the integron of SGI1-*Pm*GUE, this integron harbored the SGI1-L integron (Fig. 2, green) and the complete resistance region of SGI1-K (blue). Therefore, we named it SGI1-LK1. It also contains part of *tnpM* from Tn*21* next to the internal inverted repeat (IR) of the integron (IRi) and a Tn*4352* element (orange). However, in SGI1-LK1, a segment is inverted, and an additional IS*26* is present.

This new integron variant can be generated from the SGI1-*Pm*GUE integron via an intramolecular replicative transposition event catalyzed by IS*26* (circled in red in Fig. 2) and targeting IS*6100.* This event inverts a 19.5-kb segment and is characterized by the presence of the new IS*26* in an orientation opposite that of the IS*26* mediating the inversion and an inverted 8-bp duplication of the target site (CCGTGGGG) in IS*6100*.

### SGI1-LK1 is mobilizable by an IncC plasmid.

The standard HKL deletion (*traN* to S009) does not affect mobilization (6), nor does a longer deletion (*traN* to S0012) that inactivates or deletes all three *tra* genes (4). To determine whether the extended deletion from within *traN* to within S013 had an impact on the mobilization of SGI1-LK1 by IncC plasmids, mobilization assays were performed. SGI1-LK1 was transferred from *Pm*294MATLI containing the IncC plasmid pEA409TEM24 to Escherichia coli UB1637rif (resistant to rifampin), and the IncC plasmid pEA409TEM24 was then introduced into the transconjugant UB1637rif with SGI1-LK1. The transfer of SGI1-LK1 from this strain (UB1637rif SGI1-LK1/pEA409TEM24) to E. coli UB5201 (nalidixic acid resistant) occurred at a frequency of 6.0 × 10^−3^ transconjugants/donor (average from three independent determinations).

### Characterization of the SGI1 variant in *Pm*144BOUSA.

The P. mirabilis strain *Pm*144BOUSA was collected from a human stool specimen in 2015 at the University Hospital of Dijon. This strain was resistant to penicillins and intermediate to penicillin–β-lactamase inhibitor combinations due to the presence of the *bla*_TEM-1b_ gene. Intermediate resistance to imipenem is probably associated with alterations in penicillin binding proteins leading to a low affinity for imipenem (28), and resistance to doxycycline and colimycin is intrinsic. It was also resistant to sulfonamides and trimethoprim, and the *sul1* and *dfrA15* resistance genes were found in the draft genome. It was resistant to fluoroquinolones, and appropriate S83I and S80I alterations in *gyrA* and *parC* genes, respectively (29), were detected.

**FIG 3:**
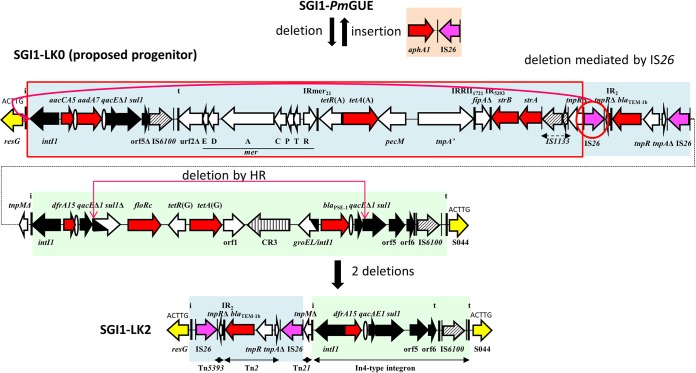
The MAR region of SGI1-LK2 and its generation from the MAR region of SGI1-*Pm*GUE and/or of the proposed progenitor SGI1-LK0. Horizontal arrows indicate genes and ORFs with their transcriptional orientation. The segments with genes and ORFs present in SGI1-K are in blue, those present in SGI1-L are in green, and those corresponding to IS*26-aphA1* are in orange. Backbone genes are represented in yellow. Black arrows represent 5′ conserved segments and 3′ conserved segments of integrons. The thick vertical bars indicate the IRs of integrons (IRi) and transposons (IRt). Resistance genes are shown with red arrows, and ovals represent *attC* sites. The ISs are represented by hatched arrows and by purple arrows for IS*26*. The IS*26*s predicted to cause rearrangements are circled. Their IRs are represented by thin vertical bars. HR, homologous recombination.

A 35.41-kb SGI1-HKL variant found at the 3′-end of the chromosomal *trmE* gene of *Pm*144BOUSA (GenBank accession number MN167852) was assembled from four contigs of the draft genome. The backbone had the alteration characteristic of the SGI1-HKL group, and the 9.57-kb integron was located at the same position as in SGI1. The integron was bounded by IRi on the left-hand side and an In4-type integron that contained the *dfrA15* cassette (like SGI1-L) on the right-hand side, with the inverted repeat of the transposon (IRt) at the right end (Fig. 3). Parts of the SGI1-K integron (blue) containing the IS*26* composite transposon with a partial *tnpR* gene of Tn*5393* and a partial Tn*2* with the *bla*_TEM-1b_ gene was also present. Therefore, this new variant was named SGI1-LK2.

SGI1-LK2 can be generated from a hypothetical progenitor of SGI1-*Pm*GUE, called SGI1-LK0, that does not harbor the IS*26-aphA1* segment found in SGI1-*Pm*GUE. SGI1-LK0 could have lost this segment by homologous recombination between the two IS*26*s forming Tn*4352*, or SGI1-*Pm*GUE could have acquired the translocatable unit (*aphA1*-IS*26*) by a conservative reaction (25). Two more steps that could have occurred in either order are needed to form SGI1-LK2 from SGI1-LK0. The deletion of most of the SGI1-K segment from SGI1-LK0 could have been promoted by an intramolecular replicative transposition of an IS*26*. Homologous recombination between the *qacE*Δ*1-sul1* regions could explain the loss of the central part of the complex class 1 integron of SGI1-L.

### Generation of SGI1-K from SGI1-LK0.

**FIG 4:**
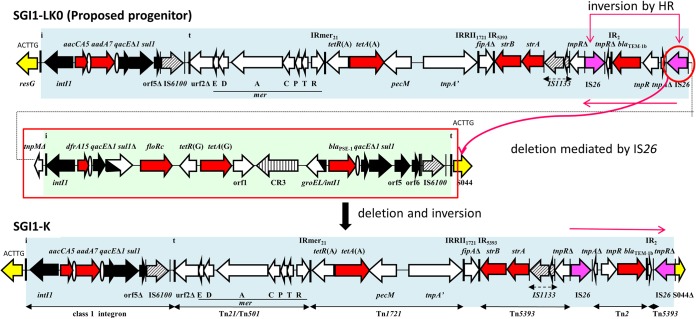
The MAR region of SGI1-K and its generation from the MAR region of the proposed progenitor SGI1-LK0. Horizontal arrows indicate genes and ORFs with their transcriptional orientation. The segments with genes and ORFs present in SGI1-K are in blue, those present in SGI1-L are in green, and those corresponding to IS*26-aphA1* are in orange. Backbone genes are represented in yellow. Black arrows represent 5′ conserved segments and 3′ conserved segments of integrons. The thick vertical bars indicate the IRs of integrons (IRi) and transposons (IRt). Resistance genes are shown with red arrows, and ovals represent *attC* sites. The ISs are represented by hatched arrows and by purple arrows for IS*26*. The IS*26*s predicted to cause rearrangements are circled. Their IRs are represented by thin vertical bars. HR, homologous recombination.

The resistance region of SGI1-K can also be generated from SGI1-LK0 in two ways. The first is via a deletion event and an inversion event (Fig. 4). The deletion of the SGI1-L complex integron, the partial *tnpM* gene, and 292 bp of the 5′ end of S044 might have been mediated by IS*26* intramolecular replicative transposition. The inversion of the segment containing a partial Tn*2* and a tiny part of Tn*5393* could have occurred by homologous recombination between the two IS*26*s framing this segment in SGI1-LK0. The inversion or deletion events could have occurred in either order.

In the other potential route for the generation of SGI1-K, the first IS*26* could have mediated a replicative transposition targeting S044 that provoked an inversion. Next, the second IS*26* of SGI1-LK0 could have promoted the deletion of the SGI1-L complex integron due to IS*26* intramolecular replicative transposition.

### Relationship between SGI1-*Pm*GUE and the SGI1-H and SGI1-L variants.

SGI1-*Pm*GUE could have arisen from SGI1-H in two steps. The first involves the insertion of the hybrid (Tn*501*/Tn*21*) *mer* module and fragments of transposons present in the SGI1-*Pm*GUE and SGI1-K variants and an In4-type class 1 integron containing the *dfrA15* cassette. These additions led to the proposed progenitor SGI1-LK0. Next, SGI1-LK0 acquired the translocatable unit *aphA1*-IS*26* and generated SGI1-*Pm*GUE.

**FIG 5:**
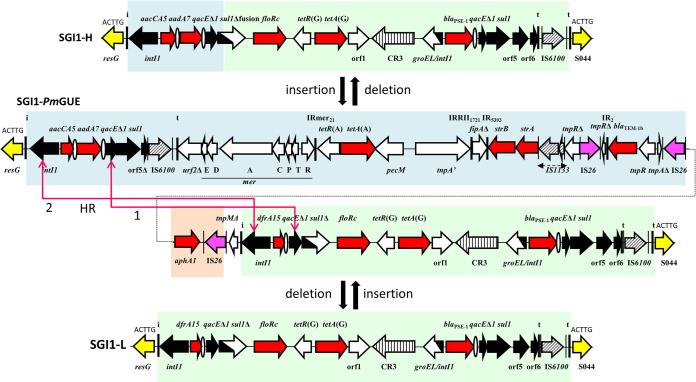
Relationship between the MAR regions of SGI1-H and SGI1-L variants and the MAR region of SGI1-*Pm*GUE. Horizontal arrows indicate genes and ORFs with their transcriptional orientation. The segments with genes and ORFs present in SGI1-K are in blue, those present in SGI1-L are in green, and those corresponding to IS*26-aphA1* are in orange. Backbone genes are represented in yellow. Black arrows represent 5′ conserved segments and 3′ conserved segments of integrons. The thick vertical bars indicate the IRs of integrons (IRi) and transposons (IRt). Resistance genes are shown with red arrows, and ovals represent *attC* sites. The ISs are represented by hatched arrows and by purple arrows for IS*26*. Their IRs are represented by thin vertical bars. HR, homologous recombination.

SGI1-L could have been created from SGI1-LK0 or SGI1-*Pm*GUE after the deletion of its central region by homologous recombination between two of the *intI1* genes of SGI1-*Pm*GUE (Fig. 5). In a similar way, SGI1-H could also be remade by homologous recombination between the *qacE*Δ*1-sul1* regions of SGI1-*Pm*GUE.

## DISCUSSION

There is a strong relationship between the members of the SGI1-HKL group, as defined previously (1). Members of this group share a characteristic alteration in the SGI backbone (13), and examination of the variation in their resistance regions highlights the extent to which changes occur within antibiotic resistance islands that are located in a specific position in a specific backbone and were likely acquired only once. The ongoing evolution of resistance islands that remain *in situ* is underappreciated, although it leads to extensive variation in the conferred resistance profile and allows the addition of genes conferring resistance to critical antibiotics such as third-generation cephalosporins to regions that already include genes conferring resistance to other potentially useful antibiotics.

**FIG 6:**
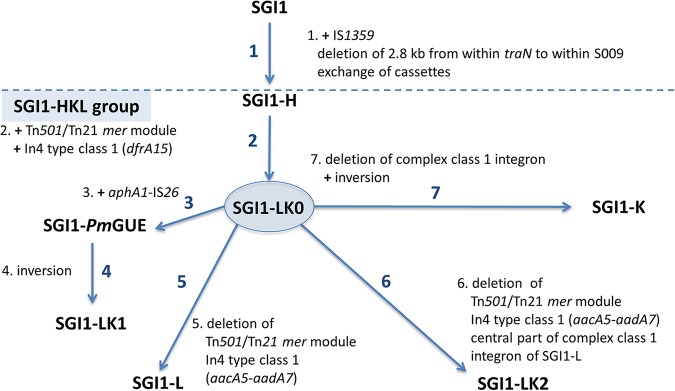
Relationship between SGI1 and variants of the SGI1-HKL group via the proposed progenitor SGI1-LK0. Steps leading to the formation of SGI1-LK0, the putative progenitor of the SGI1-HKL group, and then to new forms of the class 1 integron in the SGI1-HKL group are shown as arrows, with a summary of the events needed listed adjacent. The order of the steps is arbitrary. Details of the generation of the variants are shown in Fig. 1 to 5.

Here, SGI1-*Pm*GUE helped us to understand the generation of the other variants, such as the two new variants SGI1-LK1 and SGI1-LK2 (Fig. 6). However, the absence of the *aphA1*-IS*26* genes in SGI1-K and SGI1-LK2 suggests an alternate progenitor, SGI1-LK0, that may also be the progenitor of SGI1-*Pm*GUE. Both SGI1-LK0 and SGI1-*Pm*GUE could have arisen from SGI1-H. This hypothesis is supported by the fact that the integron of SGI1-H (SGI1-*Pm*SCO) and its derivatives SGI1-*Pm*ABB and SGI1-*Pm*MAT have already been reported in P. mirabilis (27). Hence, SGI1-*Pm*ABB and SGI1-*Pm*MAT, which have lost the central part of the complex class 1 integron of SGI1-H, can be generated by homologous recombination between *qacE*Δ*1-sul1* regions of SGI1-H.

The SGI1-L1 and -L2 variants found in *S.* Newport (18) may also have been generated from SGI1-LK0 by an IS*26*-mediated event targeting the backbone *res* gene for SGI1-L1 and movement of an IS*26*-bounded segment into S024 of the backbone for SGI1-L2. However, as for most of the SGI1 variants reported to date, these two variants have been assembled only by PCR mapping, and very limited sequence data are available, so the details of their structures cannot be examined.

The presence of IS*26* in the resistance region of most SGI1-HKL variants increases their plasticity (23–25). IS*26* can promote the insertion of new resistance genes or new structures into resistance regions (23), as for *aphA1* in SGI1-*Pm*GUE or for *bla*_CTX-M-15_ previously reported in SGI1-K7 (19). Also, the SGI1-L2 variant highlights the fact that the presence of IS*26* in these variants can promote the spread of multiply antibiotic-resistant regions to other locations. The success of the multiply antibiotic-resistant worldwide clone *S.* Kentucky ST198 (20, 26, 30) can be partly explained by the presence of SGI1-K. This clone emerged in Egypt in 1989 following the acquisition of SGI1-K and in the 1990s spread in Africa (1, 20, 26) and then in the Middle East, Asia, and European countries through different sources of contamination (20, 26, 30, 31).

The presence of SGI1-HKL variants in different serovars of S. enterica (18) and in other species (P. mirabilis [19, 27; this study] and M. morganii [22]) shows that they can be horizontally transferred even if there is a deletion from within *traN* to within S009. It has already been reported that these genes are not essential for the mobilization of SGI1 in the presence of an IncA or IncC plasmid (6, 7). The variant SGI1-LK1, which harbors a larger deletion from within *traN* to within S013, is still transferred into E. coli, and the transfer rate of SGI1-LK1 was similar to those described previously (4). This result is in accordance with previous work that showed that the transfer frequency is not significantly influenced even if there is a deletion from *traN* to S012, including *traG* and *traH* (7). However, it has been observed that both *traG* and *traH* are required for optimal transfer and that *traG* is crucial to escape from the IncC entry exclusion mechanism (32).

The SGI1-LK variants that play a key role in antimicrobial resistance in both S. enterica and P. mirabilis clearly came from a common ancestor, which had IS*1359* and the associated deletion in the backbone. The variant SGI1-H could be the original simple form from which the LK group arose as a consequence of a single insertion via homologous recombination of the segment carrying the additional region seen in the proposed progenitor SGI1-LK0. The acquisition of IS*26* and an additional integron with a third cassette array between the outer IRi and IRt of the large complex integron of SGI1-LK0 made the SGI1-LK group prone to variability. IS*26*-mediated insertions, deletions, and inversions and changes that can occur via homologous recombination within the conserved segments of the class 1 integron or between directly or inversely oriented copies of IS*26* all contribute to the creation of variant forms. This study highlights the high plasticity of the MAR region of the SGI1-LK group and shows how readily bacteria can adapt to environments subject to new antibiotic selection pressures.

## MATERIALS AND METHODS

### Bacterial isolates.

Two multiply antibiotic-resistant P. mirabilis isolates were recovered in 2015 from clinical human samples in two French hospitals in the context of our continuous study on SGI1 and *Proteus* genomic island 1 (PGI1) in P. mirabilis. DNA from the P. mirabilis isolates was extracted by heat lysis of cells and screened by PCR for the presence of the antitoxin gene *sgiA* (S026), which is present in both SGI1 and PGI1, using primers and conditions previously reported (33). The first strain, *Pm*294MATLI, was identified using matrix-assisted laser desorption ionization–time of flight (MALDI-TOF) mass spectrometry on a MALDI Biotyper (Bruker Daltonics). The other strain, *Pm*144BOUSA, was identified using the ApiE 20E system (bioMérieux, Marcy l’Étoile, France). Antibiotic susceptibility tests were performed in accordance with the guidelines of the French Committee on Antimicrobial Susceptibility Testing (Comité de l’Antibiogramme de la Société Française de Microbiologie [CA-SFM])/European Committee on Antimicrobial Susceptibility Testing (EUCAST) (https://www.sfm-microbiologie.org/) for the following antimicrobial agents: amoxicillin, amoxicillin plus clavulanic acid, ticarcillin, ticarcillin-clavulanic acid, piperacillin, piperacillin plus tazobactam, cefotaxime, ceftazidime, cefepime, aztreonam, imipenem, nalidixic acid, ofloxacin, chloramphenicol, kanamycin, spectinomycin, streptomycin, tobramycin, amikacin, gentamicin, sulfonamides, trimethoprim, and doxycycline.

### Genome sequencing and assembly.

Bacterial DNA was extracted and quantified and genome sequencing was performed on the Illumina MiSeq platform as previously reported (19). The reads were assembled using SPAdes (34) via the PATRIC Web-based platform (https://www.patricbrc.org). The contigs of the *Pm*294MATLI and the *Pm*144BOUSA draft genomes were analyzed with the Basic Local Alignment Search Tool (BLAST) (https://blast.ncbi.nlm.nih.gov/Blast.cgi). They were aligned to the complete sequence of SGI1 (GenBank accession number AF261825) to retrieve the backbone of the SGI1 variant and to the P. mirabilis HI4320 genome (GenBank accession number AM942759) to exclude the contigs belonging to the genome of P. mirabilis. ResFinder (https://cge.cbs.dtu.dk/services/ResFinder/) was used to identify contigs with acquired antimicrobial resistance genes. PCR linkage between genes belonging to nonrepeated genetic elements and Sanger sequencing were performed to close the gaps and to check the single nucleotide polymorphisms (SNPs) not present in SGI1, as previously reported (33). To ensure the correct assembly of segments flanked by IS*26*, all possible combinations were examined, and a long elongation step (7 min) was done during PCR.

### Analysis of the SGI1-HKL group.

The sequences of the new variants were compared to the complete sequences of SGI1-H (SGI1-*Pm*SCO) (GenBank accession number JX121639), SGI1-K (GenBank accession number AY463797), SGI1-L (GenBank accession number LT630458), and SGI1-*Pm*GUE (GenBank accession number JX121641) using BLAST (https://blast.ncbi.nlm.nih.gov/Blast.cgi), followed by detailed manual inspection of the outputs.

### Mobilization experiments.

The plasmid pEA409TEM24 (GenBank accession number MG764534), originally present in Enterobacter aerogenes EA409 (5) and harboring the resistance genes *bla*_TEM-24_, *aacA4*, *dfrA1*, *aadA1*, and *sul1*, was used for the mobilization assay of SGI1-LK1. For each conjugation assay, the donor and recipient strains listed in Table 1 were previously grown overnight separately in Luria-Bertani (LB) broth at 37°C at 200 rpm, with antibiotic selection in order to keep the IncC plasmid pEA409TEM24 and/or the SGI1-LK1 variant. Ceftazidime at 4 mg/liter was used to select for pEA409TEM24; chloramphenicol at 25 mg/liter or tetracycline at 10 mg/liter was used to select for SGI1-LK1; and rifampin at 50 mg/liter, nalidixic acid at 25 mg/liter, and tetracycline at 10 mg/liter were used to select E. coli UB1637rif (resistant to rifampin), E. coli UB5201 (resistant to nalidixic acid), and P. mirabilis *Pm*294MATLI (intrinsically resistant to tetracycline), respectively (Table 1).

**TABLE 1:** Donor and recipient strains with their resistance profiles and antibiotic selection used for culture overnight before the conjugation assay

Donor or recipient strain	Resistance profile^*a*^
E. coli UB5201	NAL
E. coli UB5201 SGI1-LK1	NAL, **AMP, CHL, KAN, GEN, STR,** **SUL, TMP, TET**
E. coli UB5201/pEA409TEM24	NAL, **AMP, SAM, CAZ, KAN, AMK,** **STR, SUL, TMP**
E. coli UB1637rif	RIF
E. coli UB1637rif SGI1-LK1	RIF, **AMP, CHL, KAN, GEN, STR,** **SUL, TMP, TET**
E. coli UB1637rif SGI1-LK1/pEA409TEM24	RIF, **AMP, CAZ, CHL, KAN, GEN,** **AMK, STR, SUL, TMP, TET**
P. mirabilis *Pm*294MATLI(SGI1-LK1)	**AMX, CHL, KAN, GEN, SPT, STR,** **SUL, TMP, TET**
P. mirabilis *Pm*294MATLI(SGI1-LK1)/pEA409TEM24	**AMP, SAM, CAZ, CHL, KAN, GEN,** **AMK, STR, SUL, TMP, TET**

a Antimicrobial resistance conferred by pEA409TEM24 or SGI1-LK1 is shown in boldface type. Antimicrobial susceptibility tests were performed in accordance with Clinical and Laboratory Standards Institute (CLSI) guidelines except for the P. mirabilis *Pm*294MATLI(SGI1-LK1) strain, which was previously tested in accordance with the guidelines of the French Committee on Antimicrobial Susceptibility/European Committee on Antimicrobial Susceptibility Testing (CA-SFM/EUCAST). The underlined antibiotics were used to keep the pEA409TEM24 plasmid and SGI1-LK1 and to select the strain. NAL, nalidixic acid; AMP, ampicillin; CHL, chloramphenicol; KAN, kanamycin; GEN, gentamycin; STR, streptomycin; SUL, sulbactam; TMP, trimethoprim; TET, tetracycline; SAM, ampicillin-sulbactam; CAZ, ceftazidime; AMK, amikacin; RIF, rifampin; AMX, amoxicillin; SPT, spectinomycin.

Conjugation assays were performed by mixing equal amounts of the donor and recipient and growing them overnight at 37°C on LB agar, as previously reported (6). The plasmid pEA409TEM24 was transferred from UB1637rif into *Pm*294MATLI(SGI1-LK1) [harboring the resistance genes *aacCA5*, *aadA7*, *sul1*, *tetA*(A), *strAB*, *bla*_TEM-1b_, *aphA1*, *dfrA15*, *floR*, and *blaP*], and *Pm*294MATLI(SGI1-LK1) containing pEA409TEM24 was recovered on selective medium with chloramphenicol and ceftazidime (Table 2). Next, SGI1-LK1 from *Pm*294MATLI with pEA409TEM24 was transferred into E. coli UB1637rif. The transconjugant UB1637rif with SGI1-LK1 was selected with rifampin and chloramphenicol. Next, pEA409TEM24 was transferred from UB5201 into UB1637rif containing SGI1-LK1. UB1637rif SGI1-LK1 containing pEA409TEM24 was selected with tetracycline and ceftazidime. Finally, three mating assays were performed independently between UB1637rif SGI1-LK1/pEA409TEM24 and UB5201. The donor strain UB1637rif was selected with rifampin, and the recipient strain UB5201 with SGI1-LK1 was selected with nalidixic acid and tetracycline. The transfer frequency of SGI1-LK1 was calculated as the number of transconjugants per donor.

**TABLE 2:** Mating assays

Purpose of mating assay	Donor	Recipient	Selection, medium^*a*^	Transconjugant
Transfer of pEA409TEM24 into *Pm*294MATLI	UB1637rif/pEA409TEM24	*Pm*294MATLI (SGI1-LK1)	CHL + CAZ, MacConkey	*Pm*294MATLI (SGI1-LK1)/pEA409TEM24
Transfer of SGI1-LK1 into *E. coli* UB1637rif	*Pm*294MATLI (SGI1-LK1)/pEA409TEM24	UB1637rif	RIF + CHL, MacConkey	UB1637rif SGI1-LK1
Transfer of pEA409TEM24 into *E. coli* UB1637rif SGI1-LK1	UB5201/pEA409TEM24	UB1637rif SGI1-LK1	RIF + TET + CAZ, LBA	UB1637rif SGI1-LK1/pEA409TEM24
Measurement of transfer rate	UB1637rif SGI1-LK1/pEA409TEM24	UB5201	RIF (donor) and NAL + TET (transconjugant), LBA	UB5201 SGI1-LK1

a The following antibiotics were used: nalidixic acid (NAL) (25 mg/liter) for selecting UB5201, rifampin (RIF) (50 mg/liter) for selecting UB1637rif, chloramphenicol (CHL) (50 mg/liter) or tetracycline (TET) (10 mg/liter) for selecting SGI1-LK1, and ceftazidime (CAZ) (4 mg/liter) for selecting the plasmid pEA409TEM24. LBA, Luria-Bertani agar medium.

To check for the presence of SGI1-LK1 and the IncC plasmid in the transconjugants, antibiotic susceptibility tests were performed in accordance with Clinical and Laboratory Standards Institute (CLSI) guidelines, and PCRs were performed as previously described (6), with the same primers. For P. mirabilis *Pm*294MATLI, the presence of the *sgiA* gene (S026) was screened to confirm the presence of the SGI1-LK1 variant using the same primers as the ones previously reported (27).

### Accession number(s).

The sequences of SGI1-LK1 and SGI1-LK2 were submitted to GenBank under accession numbers MH734354 and MN167852, respectively.
