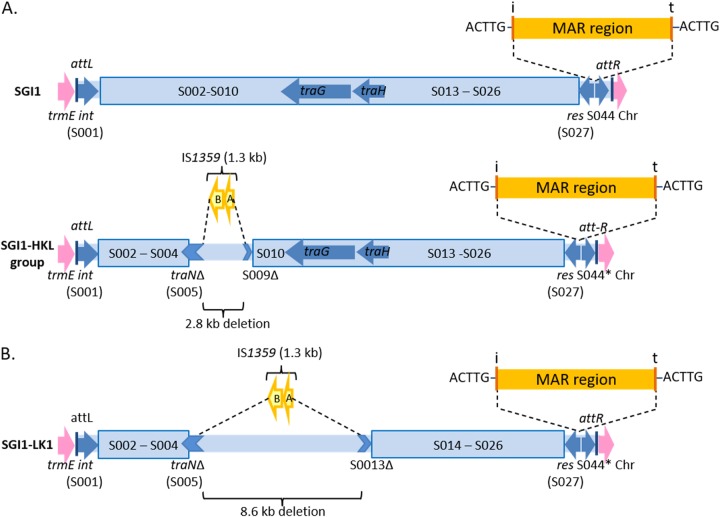# Erratum for de Curraize et al., “Two New SGI1-LK Variants Found in Proteus mirabilis and Evolution of the SGI1-HKL Group of *Salmonella* Genomic Islands”

**DOI:** 10.1128/mSphere.00435-20

**Published:** 2020-05-13

**Authors:** Claire de Curraize, Eliane Siebor, Véronique Varin, Catherine Neuwirth, Ruth M. Hall

**Affiliations:** a Bacteriology Department, University Hospital Dijon, Dijon, France; b UMR 6249, Chrono-Environnement, Dijon, France; c School of Life and Environmental Sciences, The University of Sydney, Sydney, New South Wales, Australia

## ERRATUM

Volume 5, no. 2, e00875-19, 2020, https://doi.org/10.1128/mSphere.00875-19. Figure 1: in the original published version, the *traG* and *traH* genes of SGI1 were depicted in the wrong orientation. The correct version is shown below.

**FIG 1.**